# Acute early transplant renal artery thrombosis; a complex etiologic diagnosis

**DOI:** 10.12860/jnp.2014.28

**Published:** 2014-10-01

**Authors:** Alireza Hamidian Jahromi, Bahar Bastani

**Affiliations:** ^1^Department of Surgery, Louisiana State University Health Sciences Center, Shreveport, Louisiana, United States; ^2^Division of Nephrology, Department of Medicine, Saint Louis University School of Medicine, Saint Louis, Missouri, United States

**Keywords:** Transplantation, End stage renal failure, Renal replacement therapy, Organ transplant, Transplant renal artery stenosis, Transplant renal artery thrombosis

**Implication for health policy/practice/research/medical education:**

Vascular complications are the most dreaded surgical complications in renal transplantation and can cause sudden renal allograft loss. Vascular complications are reported in 1-3% of the renal transplant recipients, and among them transplant renal artery stenosis and thrombosis compromise 50-80% of the complications.

## Sir,

We read with great interest the report published by Fallahzadeh *et al*. on their experience with a female recipient of a deceased donor renal transplant who presented with a transplant renal artery thrombosis one week after the operation (1). However, we have a few queries that we would like to get clarified by the authors.

Vascular complications are the most dreaded surgical complications in renal transplantation and can cause sudden renal allograft loss. Vascular complications are reported in 1-3% of the renal transplant recipients, and among them transplant renal artery stenosis and thrombosis compromise 50-80% of the complications (2,3).

First, the authors did not mention whether their patient received any antibody induction. As shown in an earlier case report certain antibody inductions can result in transplant renal artery thrombosis, presumably secondary to a transient hypercoagulable state from cytokine release (4).

Second, we would like to know whether this patient was on aspirin or any other anti-coagulation medication when she was discharged home (six days post operation) or at the time of presentation to the hospital with decreased urine output leading to a diagnosis of renal artery thrombosis (seven days post operation).

Third, while we agree with the management plan of the authors in this case, using catheterization of the occluded artery and subsequent thrombectomy, we would like to ask the authors to explain which radiologic angiography characteristics helped them in the differentiation between a retained clot, a ruptured atheroma, and distal renal artery stenosis. There are also reported cases where fibromuscular hyperplasia has been considered as a potential cause for transplant renal artery stenosis and thrombosis (5). Differentiating fibromuscular hyperplasia from other causes of transplant renal artery stenosis based on the radiologic evaluation may not be an easy call.

Fourth, was the patient started on heparin infusion following the thrombectomy and stent placement? Was the activated partial thromboplastin time (aPTT) within the therapeutic range during the first 24 hours after thrombectomy and when the patient formed renal graft thrombosis for the second time? Did the patient have any episodes of hypotension in the post-operative period or after the initial attempt at thrombectomy and stent placement?

Fifth, severe renal artery stenosis (either in the native or the allograft kidney) is a well-known cause of arterial hypertension and renal insufficiency (6). While the beneficial effect of percutaneous transluminal renal angioplasty and stenting in treating transplant renal artery stenosis has been shown before (6-9), the preference for different types of stents in cases with renal artery thrombosis complicating the stenosis has not been discussed in detail. We would like to ask the authors to clarify what type of stent they used in the current case report?

Sixth, we would like some clarification as to whether thrombophilia screening and tests, such as protein C, protein S, antithrombin III deficiency, factor V Leiden mutation, prothrombin mutation and mutation in the *MTHFR* gene, were done in this recipient before or after the episode of allograft thrombosis, or in the acute phase when the patient was readmitted to the hospital? The authors have mentioned that the hypercoagulability work up was unremarkable, but they did not list the tests they used for the hematologic evaluations (or the depth of their investigations). Did the patient have any history of thrombosis in the past?

Seventh, the time elapsed between the transplantation and the graft thrombosis (immediate, early, or late thrombosis) can sometimes help in differentiating the cause for thrombosis in the grafts. Considering the timing of the thrombotic event in the current case, it would be challenging to blame the preexisting transplant renal artery stenosis as the only underlying cause for the graft thrombosis. One would expect such cases to present within the first 24 hours after transplantation. We would appreciate if the authors would comment on this speculation.

We would like to thank the authors for this interesting case report highlighting the importance and the challenging situation of thrombotic transplant renal artery event. We share the view and agree that this complication should be considered as an important differential diagnosis in the post-transplant patients presenting with anuria or severe oliguria, deterioration of kidney function and pain at the site of the graft. An in depth investigation for the possible underlying factors is always warranted which is the cornerstone for the prevention of similar events to the transplanted organ in case future transplants would be considered in the patient.

## Authors’ contributions

All authors wrote the paper equally.

## Conflict of interests

The authors declared no competing interests.

## Funding/Support

None.
